# A case report of simultaneous tears of the medial and lateral posterior meniscal roots with an intact ACL graft

**DOI:** 10.1016/j.ijscr.2024.109971

**Published:** 2024-06-29

**Authors:** Nasser Sulaiman Alsaleh, Abdullah Adel Alnasser, Abdulaziz Ali Alqahtani

**Affiliations:** a Prince sultan military medical city, Riyadh, Saudi Arabia; b Orthopedic resident at prince Sultan military medical city, Riyadh, Saudi Arabia; c Department of Orthopedic Surgery, Prince Sultan Military Medical City, Riyadh, Saudi Arabia

**Keywords:** ACL, Meniscus, Tear, Posterior horns, Case report

## Abstract

**Introduction and importance:**

Meniscal root tears are defined as soft-tissue and/or osseous injuries that rip or avulse within one centimeter of the meniscal insertion to the tibial plateau. These injuries impact around 100,000 patients a year and make up 10 % to 21 % of all meniscal tears. Meniscal extrusion frequently happens when there are root rips, and the transmission of circumferential hoop loads is hampered.

**Case presentation:**

We present one case of a 28-year-old male who complained of pain and stiffness in his left knee since 2 years after undergoing ACL reconstruction using a hamstring autograft. His examination revealed joint line tenderness on both the medial and lateral sides of the left knee. Further investigations involving X-ray and MRI established the diagnosis of both medial and lateral meniscal root tears, which were surgically managed using the transtibial pullout technique.

**Discussion:**

The biomechanical implications of meniscal root tears, such as loss of hoop forces and increased tibiofemoral contact pressures, underscore the importance of timely diagnosis and management. The literature advocates surgical treatment for managing root tears, as leaving them without surgical intervention can lead to functional outcomes similar to those of total meniscectomy. Conclusion: This case report presents both menisci posterior root tears with an intact ACL graft which is unique in that they commonly tear in conjuction with ACL. These kind of injuries necessitates prompt diagnosis and surgical intervention to protect the knee from early arthritic changes.

## Highlights

• Meniscus tear following ACL reconstruction.
• Simultaneous medial and lateral posterior horn tear
• Outcome of meniscal repair following ACL reconstruction.

## 1 Introduction

**Fig. 1:**
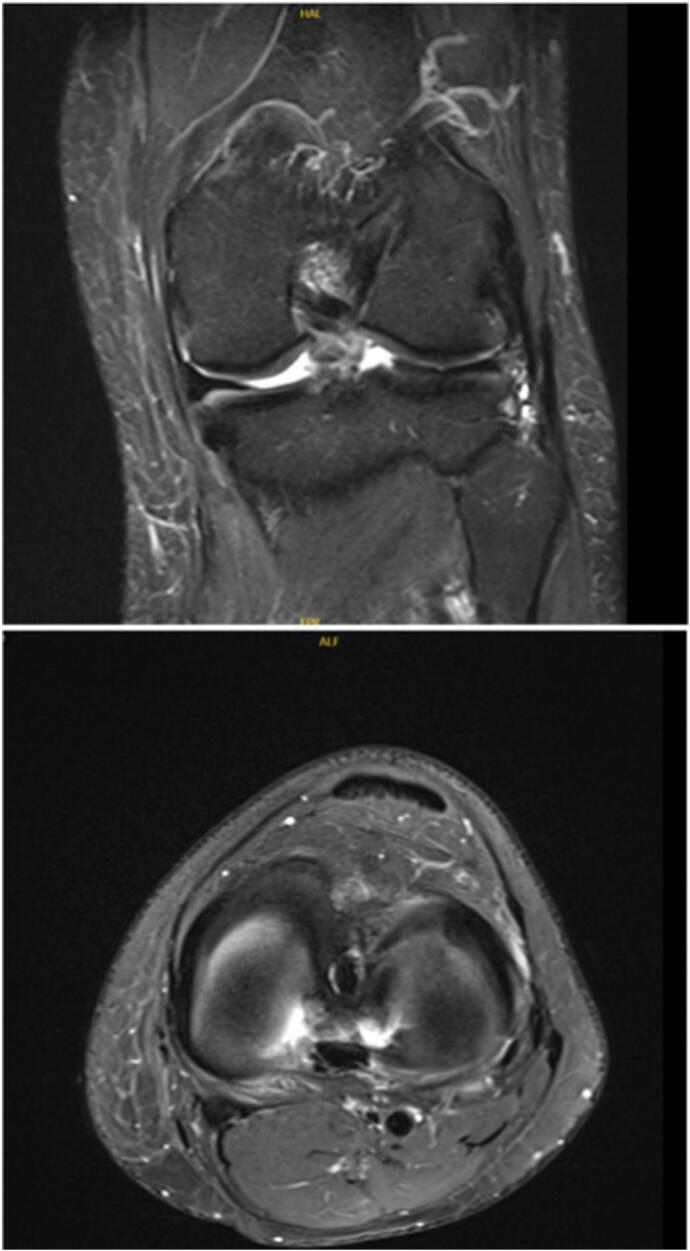
MRI (coronal and axial cuts) showing fraying and loss of meniscal tissue at the posterior horn of the medial and lateral meniscus.

Knee trauma often results in meniscal tears, with torn portions displaced from their original position. Meniscus root tears, comprising up to 10–21 % of all meniscal tears, have gained attention in recent years [1]. Meniscal root tears are characterized by either the detachment of the meniscus attachment or complete radial tears occurring within a proximity of 1 cm from the meniscus insertion [2]. The primary anchorage points of the menisci to the tibial plateau are provided by the anterior and posterior roots of both the medial and lateral menisci [3]. The tibiofemoral joint exerts axial loading, compressing and applying a radial force on the meniscus. The circumferential fibers of the meniscus resist this radial force, resulting in a circular shape. The concept of ‘hoop stress’ is integral, primarily transmitted via the meniscal roots [4]. When the meniscal root sustains damage, it results in the loss of hoop stress, leading to extrusion due to radial force exerted by axial loading. This compromises the joint's ability to absorb and distribute contact forces effectively [5]. Medial meniscus posterior root tears typically stem from chronic degenerative meniscal disease, while lateral meniscus posterior root tears are predominantly linked to traumatic events, often associated with anterior cruciate ligament injuries [6]. Literature reports the adverse effects of meniscal root tears on preserving typical knee joint mechanics, managing contact forces, and averting osteoarthritic developments [4,7]. Although meniscal root injury is not uncommon, simultaneous injury of both medial and lateral roots is rare, and our review of the literature showed only few reports on such cases [[8], [9], [10], [11]]. In this report, we present the case of a patient with simultaneous posterior root injury, with a history of ACL reconstruction two years back (Fig. 1).

**Fig. 2:**
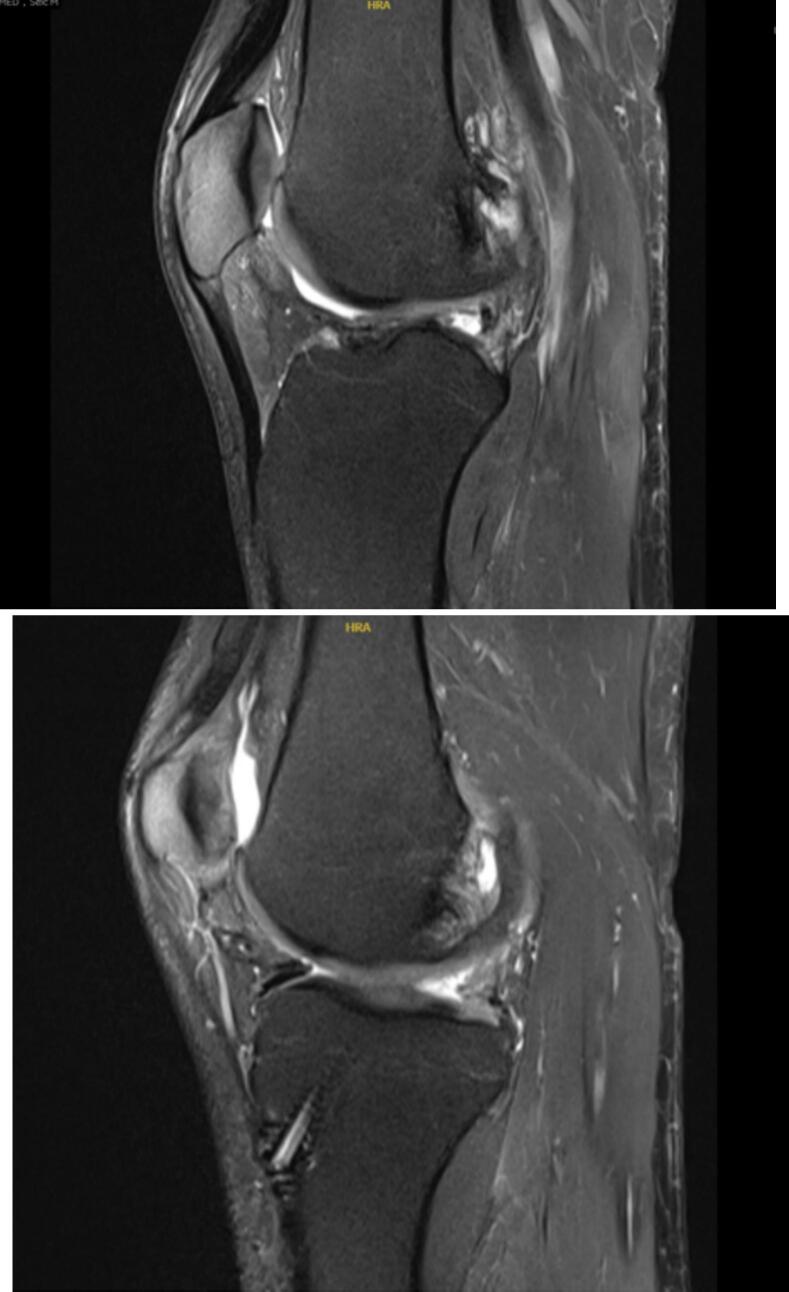
T2 magnetic resonance imaging sagittal cuts showing increased signals at the posterior roots of lateral (a) and medial (b) meniscus.

The case has been reported in line with the SCARE criteria [12] (Fig. 2).

## 2 Case report

A 28-year-old male presented with the complaint of pain and stiffness in his left knee. These symptoms started two years following successful ACL reconstruction surgery using a hamstring autograft, which was followed by a comprehensive physical therapy program. Following the initial surgery, the patient resumed regular daily activities without any limitations and engaged in sports 9 months postoperatively. Approximately one year after the initial surgery, he joined a military training program that lasted for nine months. During this training, he developed knee pain and stiffness despite the absence of any traumatic incidents. He continued training for an additional four months before seeking medical evaluation. The primary complaint was pain, without symptoms of knee instability or locking.

## 3 Clinical findings

On clinical examination, the patient displayed joint line tenderness on both the medial and lateral sides of the knee. The pivot, Lachman, and varus/valgus stress tests were negative.

## 4 Diagnostic assessment

MRI revealed marked fraying with significant loss of meniscal tissue at the posterior horn of the medial and lateral meniscus at its junction with roots in keeping with almost a full thickness radial tear (Fig. 1, Fig. 2).

## 5 Therapeutic intervention

**Fig. 3:**
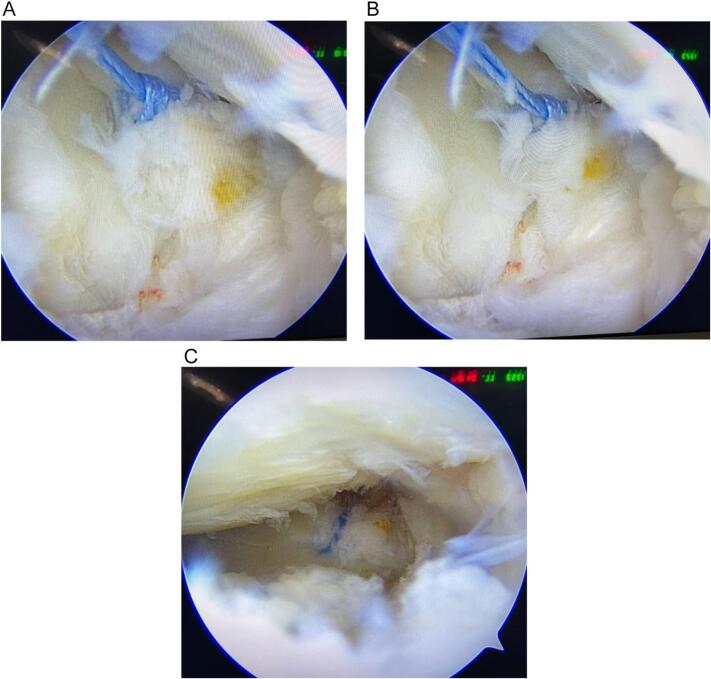
(A&B) arthroscopic images showing complete tear lateral meniscus root. C. shows post trans-tibial fixation of lateral meniscus root.

**Fig. 4:**
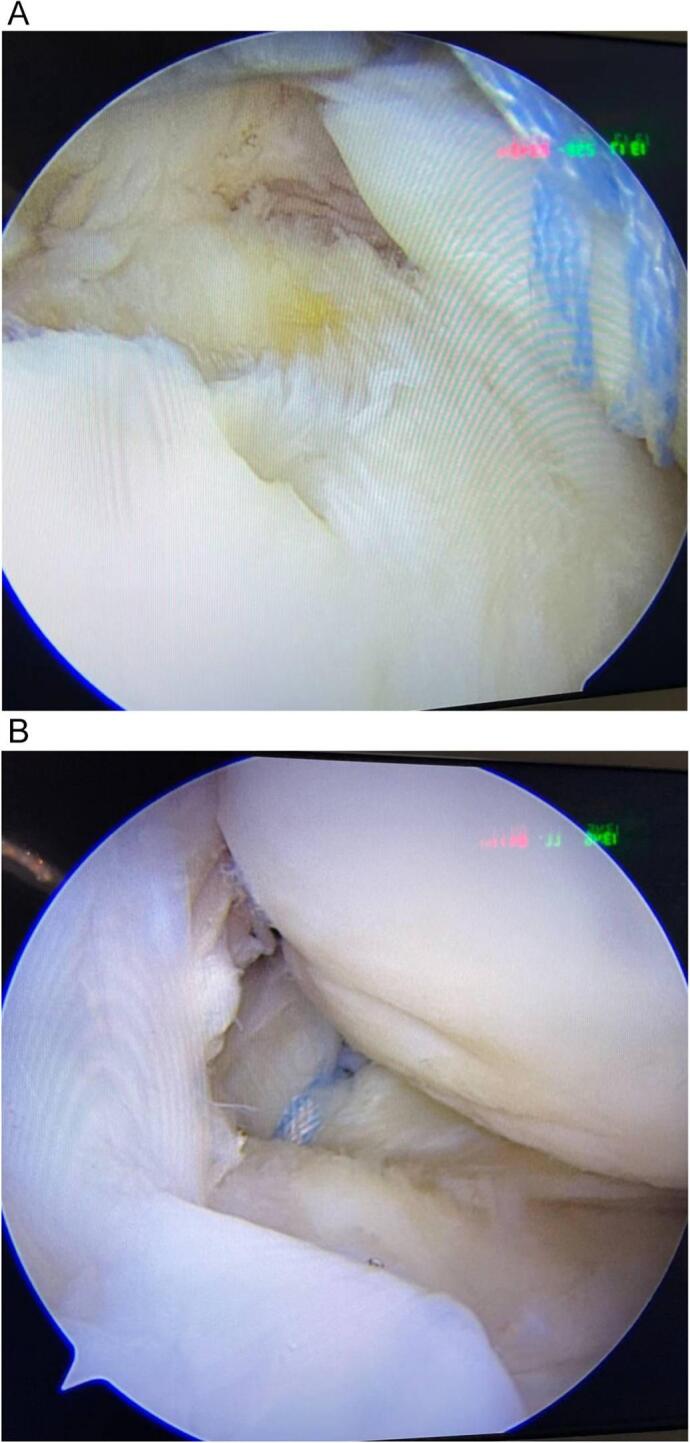
A: arthroscopic images showing complete medial meniscus root tear B: shows post trans-tibial fixation of medial meniscus root.

Arthroscopic examination was performed using anteromedial and anterolateral portals, which revealed bilateral root posterior horn tears in both menisci with intact ACL graft, and no other pathologies were identified. Fenestration was performed in the medial collateral ligament (MCL) to enhance the exposure of the medial meniscus. Preparation of the root bed was performed using a curate and shaver. Using Ultra-braid sutures #2 through a suture passer (FirstPass mini, Smith, and nephew) were taken through the meniscal roots and a transtibial tunnel fixed with *endo*-buttons for both roots. Arthroscopic assessment of both roots after fixation revealed stable meniscal roots with excellent fixation strength (Fig. 3, Fig. 4 ).

## 6 Follow-up and outcomes

The patient was instructed to remain non-weight-bearing for six weeks with 0–90 degrees knee range of motion. This was followed by full ROM exercises and full weight bearing with gradual strengthening exercises. Return to sports and cutting activities was achieved five months postoperatively.

**Fig. 5:**
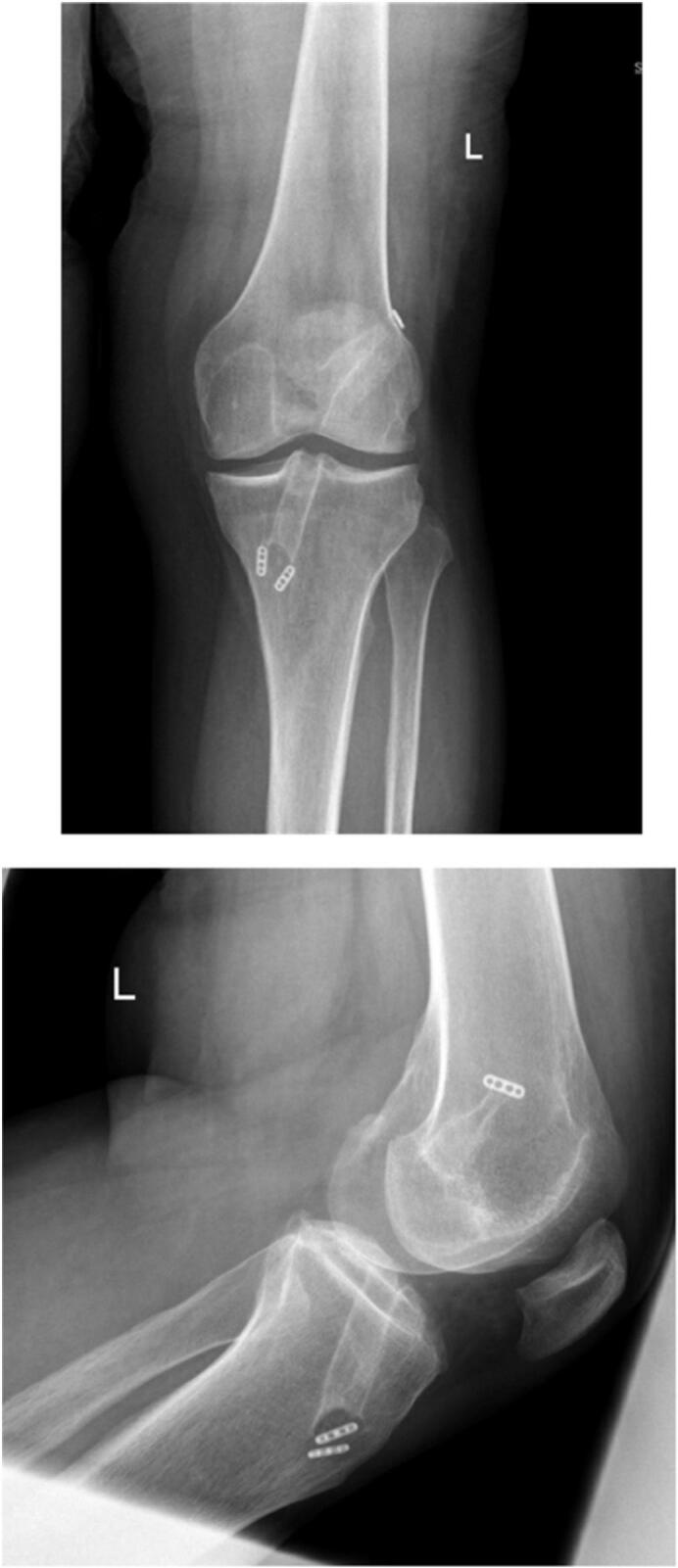
Full weight bearing radiograph of the left knee 2 years post-operatively.

The patient achieved full knee ROM, with no pain or limitations in daily activities. At the two-year follow-up, the patient reported no pain or stiffness, exhibited full knee ROM, and had a Lysholm knee score of 94 (Fig. 5).

## 7 Discussion

Meniscal extrusion is prevalent in medial meniscal root tear (MMRT) patients. It is uncommon in lateral meniscal root tear (LMRT) patients, nevertheless, as they are more frequently linked to a history of trauma and ACL tears [13]. Concomitant injury of both meniscal roots is extremely rare, and only a few reports have described such cases [[8], [9], [10]], all of which have described an associated ACL injury, and only one case has reported bilateral meniscal tear injuries in the presence of an intact ACL [9]. A LMRT incidence of 2.9 % was reported out of 559 knee MRIs by De Smet et al [14] Nonetheless, the observed incidence of LMRTs increases to 8 % in the event of an ACL tear [14].

The biomechanical implications of meniscal root tears, such as loss of hoop forces and increased tibiofemoral contact pressures, underscore the importance of timely diagnosis and management [15]. The literature advocates surgical treatment for managing posterior horn medial meniscus root tears, as leaving untreated or misdiagnosing the MMPRT can lead to functional outcomes similar to those of total meniscectomy [9]. Some studies advocate for conservative management of lateral meniscal root injuries; however, these studies were conducted on patients with an associated ACL injury that could help in the process of healing, as ACL injury has been reported to trigger certain healing factors that may help in the healing of the meniscus [16]. Other advocates for surgical treatment, a study conducted by Lee et al. shows that the peak tibiofemoral contact pressure rises from 2.8 to 4.2 MPa; however, pressure levels normalize following pullout repair via a transtibial tunnel [9]. In this case report, we present a case of medial and lateral meniscal posterior horn root tears in a patient with a history of ACL reconstruction.

## 8 Conclusion

Injury to both meniscal posterior horn roots in an intact ACL graft is rare. This case report presents a rare injury that necessitates prompt diagnosis and surgical intervention to protect the knee from early arthritic changes.

## Consent

Written informed consent was obtained from the patient for publication of this case report and accompanying images. A copy of the written consent is available for review by the Editor-in-Chief of this journal on request.

## Ethical approval

Ethical approval for this was provided by the Ethical Committee of prince sultan military medical city on 15 Sep 2023.

## Funding

We declare that we didn't receive any funds for this study.

## Author contribution

Dr. Nasser alsaleh: manuscript revision.

Dr. Abdullah alnasser: manuscript writing and drafting.

Dr. abdulaziz alqahtani: article supervision and approval.

## Guarantor

Abdullah alnasser.

## Research registration number

N/A.

## Conflict of interest statement

We (the authors) declare that we don't have any financial or personal relationships with other people or organisations that could inappropriately influence (bias) our work.
